# Influence of Mediterranean Diet and Incidence of Global Warming on Food Habits and Plant Growth in Northern Mediterranean Latitudes: Narrative Review

**DOI:** 10.3390/nu17040677

**Published:** 2025-02-14

**Authors:** Norbert Latruffe, Gérard Lizard

**Affiliations:** 1 Mediterranean Diet and Health Association (NMS), Center of Taste and Food (CSGA UMR UB-CNRS-INRAe-Institut Agro), Laboratory Bio-PeroxIL, University of Burgundy Europe, 21000 Dijon, France; 2 Mediterranean Diet and Health Association (NMS), INSERM (Institut National de la Santé et de la Recherche Médicale), Laboratory Bio-PeroxIL, University of Burgundy Europe, 21000 Dijon, France; gerard.lizard@u-bourgogne.fr

**Keywords:** climate change, global warming, Mediterranean diet, food habits, farming, Northern Mediterranean latitudes, Burgundy

## Abstract

Background: Climate change has consequences for farming, food diversity and availability, and diet habits. There is now evidence that the Mediterranean climate is rapidly spreading to the Northern European latitudes. Objective: This narrative review aims to identify relevant studies related to climate change that could favor the progression of the Mediterranean climate in the northern latitudes of Europe, mainly in France, and to predict what the consequences of these changes on the human diet could be, especially using the concept of the Mediterranean diet, with subsequent impacts on health, farming, and eating habits. Methods: This narrative review was realized by consulting the PubMed, Scopus, Science Direct, and Google Scholar databases. Results: The key points developed in this review are as follows: investigating the Mediterranean diet as a healthy diet, with evidence supporting health benefits and perspectives; similarities with other places in the world at the same Mediterranean latitudes; climate change and the resulting consequences on plant growth, farming, and food habits; and perspectives on the need for societal adaptations of populations towards agriculture, food, and cooking changes. As climate change facilitates the development of new farming practices with more or fewer environmental impacts, the growth of Mediterranean plants in the highest latitudes of Europe, such as olive trees, pomegranates, and almonds, has already begun for economic reasons. Future perspectives: In the near future, besides economic interests, climate change will favor the consumption of several products associated with the Mediterranean diet in the Northern European latitudes. In this context, producers and consumers play major roles. 
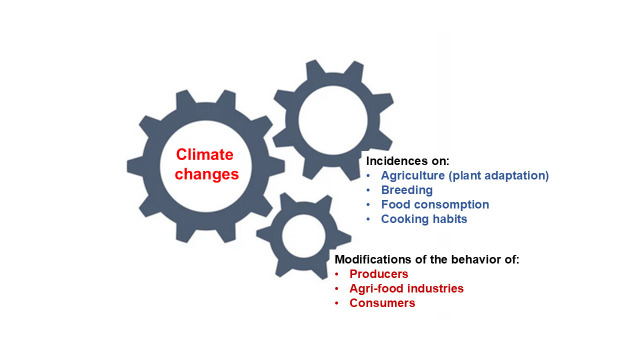

## 1. Introduction

The early history of the earth includes the succession of cooling and warming periods with major changes for life: the disappearance, survival, and evolution of plant and animal species [1,2]. For the last 1000 years, in the temperate latitudes of Western Europe, there have been cyclic changes of 150–200-year periods, and the last one started around 100 years ago. Based on the consequences of global warming, within 50 years from now, it is supposed that the Mediterranean climate, currently observed around the Mediterranean Sea, could move to higher latitudes, especially in France [3,4]. Advances in proxy methods and interpretations pave the way for the use of past climates for model evaluation [5,6]. Unfortunately, the area currently characterized by a Mediterranean climate could progressively become semi-arid and arid, with several negative social and economic impacts on the countries concerned [3]. In the South of Europe, one of the major impacts of global warming notably concerns vine and corn cultivation; indeed, vines are sensitive to dehydration and warm and sunny days, and the cultivation of corn requires a high quantity of water [4]. This impact on vine growth has already had negative economic impacts in several wine regions in France. Conversely, in the highest latitudes of Europe, this situation of global warming could favor modifications to agricultural practices, with the development of new cultures (olives, pomegranates, almonds) and new white oak forests (https://www.oliveoiltimes.com/production/climate-change-leads-some-bordeaux-wine-producers-to-plant-olives/116924, accessed on 15 January 2025), and subsequently, new food habits. This could lead to an increased consumption of fruits and vegetables that are major compounds of the Mediterranean diet. The notion of the healthy diet began to emerge from 1950 to 1990 with the work of the American physiologist Ancel Keys, who looked at men in their 40s and 50s living in Greece (including Crete), the US, Finland, Japan, Italy, Yugoslavia, and the Netherlands. Ancel Keys showed that despite a diet very rich in fat (over 40% of their daily calorie intake came from olive oil in Greece), cardiovascular diseases that resulted in heart attacks and stroke in these countries were almost unheard of, cancer was even rarer, and an increase in lifespan in good health was, therefore, observed (https://borakisgreekfood.co.uk/pages/the-cretan-diet, accessed on 21 January 2025 and [7]). This pioneering work of Ancel Keys contributed to the notion of the Mediterranean diet and to the emergence of the work of Serge Renaud on the French paradox [8]. It is now widely admitted that the Mediterranean diet has several health benefits [9], and in association with the current environmental changes that have several negative aspects [10], some modifications of diet habits, including more Mediterranean compounds (vegetables, fruits), could be expected [11]. It is important to highlight that the so-called Mediterranean style of cooking is currently not restricted to the Mediterranean basin but is also found in other regions of the globe at the same latitude, between the 39th and 40th parallels from the northern and southern hemispheres, including California (USA) and certain regions of China, Chile, and South Africa [12,13]. Currently, to improve evidence for the health benefits of the Mediterranean diet, more systematic and quantitative approaches are still needed in research practice, and the applicability of the Mediterranean diet to non-Mediterranean countries has not been established. In addition, the Mediterranean diet also includes a lifestyle aspect, requiring the simultaneous consideration of other non-dietary behavioral factors when assessing its effects [14]. 

Climate change, which is currently a dramatic situation for several countries, must be rapidly taken into consideration to transform this change into an opportunity for innovation in different fields. For example, the goal is to adapt plant species (vegetables, trees, herbs, spices) and vineyards to climate change with methods having no or few minor environmental impacts and no negative consequences on human and animal health [15,16,17]. The development of innovative methods of creating cultures without environmental impact to endorse new socio-economical concepts seems crucial [18]. The following objective will consider the advantage of keeping local production while reducing the carbon footprint produced in a circular economy [19]. An important question is how we can stimulate people to embrace this health-food approach based on the Mediterranean diet and encourage them to adopt it. In this context, some proposals have been presented at a round table organized by the «Nutrition Méditerranéenne et Santé» (NMS) association [20] on the following topic: “Which agriculture and food of the Mediterranean diet will be used in the next 50 years, in the septentrional area of France”.

The present narrative review focuses on the aspects of climate change that could favor an extension of the Mediterranean climate in the highest latitudes of Europe, especially in France, with an impact on diet habits. The key points developed in this review are as follows: healthy diet: Mediterranean (and Cretan) diet, proof of health benefit perspectives; similarities between other places in the world at the same Mediterranean latitudes; climate change and its resulting consequences on food habits and plant growth; and perspectives on the need for societal adaptations of populations towards agriculture, food, and cooking changes. To realize this narrative review, PubMed, Scopus, Science Direct, and Google Scholar databases were consulted. The methodology used for this narrative review is described in the Section 2.

## 2. Methods

**Figure 1:**
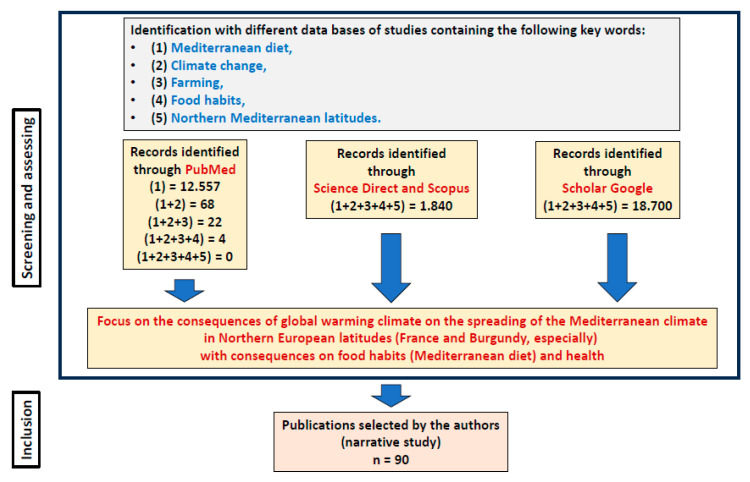
Flowchart used to select the references appropriated for this study (narrative review). Elaborated by the authors. (n = 90; 84 references (list of references) and 6 https links).

This narrative review was performed following different steps [21]. For this, PubMed, Scopus, Science Direct, and Google Scholar databases were consulted to identify the relevant studies. The final search was conducted in January 2025 and included English-language-based international articles, online reports, and electronic books. The following keywords (climate change; global warming; Mediterranean diet; food habits; farming; and Northern Mediterranean latitudes), identified in the title, abstract, and keywords of articles, were used and combined; they were also combined with economic impact, social impact, and environmental impact. After the search was complete, the abstracts were read and selected by the authors to ensure that they addressed topics of interest. All duplicates were removed by the authors, and the abstracts of the remaining articles were reviewed to ensure that they addressed the review inclusion criteria. The corresponding publications were read by the authors. As this is a narrative review, the publications were selected from the databases using the above-mentioned keywords, and those considered the most relevant were selected by the authors and cited. Figure 1 corresponds to the chart flow to elaborate on the selection of bibliography references. 

## 3. The Mediterranean Diet as a Healthy Option: Benefits and Perspectives

A diet, especially one rich in antioxidants, is the Mediterranean diet [22,23]. It is well recognized that the regular consumption of green vegetables, fruits, fibers, and fish, combined with daily exercise and social activities, has a protective effect against the onset of age-related diseases (mainly cardiovascular diseases) and, consequently, is a factor of longevity [24]. Conversely, a high-calorie diet (rich in refined sugars and fats), combined with deleterious life habits (smoking, excessive alcohol consumption, stress), will increase the risk of vascular pathologies, diabetes, obesity, and cancer [25]. In fact, plant-based foods contain several types of microconstituents present in fruits and vegetables, such as polyphenols with antioxidants, anti-aging properties [4], and phytosterols with hypo-cholesterolemic properties [26]. Examples of antioxidant-rich fruits and vegetables are grapes, tea/coffee, soy, peanuts, cocoa, apples, onions, cabbages, broccoli, tomatoes, almonds, olive oil, pomegranates, red berries (blueberries, blackcurrants, raspberries), etc. These products and some of their derivatives, including wine, juices, and/or oils, offer powerful antioxidant properties and sometimes anti-inflammatory properties. Antioxidants and anti-inflammatory compounds are extremely important to vital processes since cellular aging seems directly linked to increased levels of free radicals and inflammatory cytokines [27]. 

Currently, the Mediterranean diet is considered an intangible heritage of humanity by the United Nations Educational, Scientific and Cultural Organization (UNESCO) (https://www.unesco.org/archives/multimedia/document-1680-eng-2, accessed on 21 January 2025; and [28]). The Mediterranean diet is characterized by a nutritional model that has remained constant over time and over space, the main ingredients of which are olive oil, cereals, fruit, and vegetables, either fresh or dried, a limited proportion of fish, dairy products, and meat, and numerous condiments and spices. All are accompanied by drinks or infusions, always respecting the beliefs of each community. The Mediterranean diet is also defined as a set of skills, knowledge, practices, and traditions that go beyond vegetables and fruits, accompanied either by wine or infusions. At the moment, there are several scientific studies on the benefits of the Mediterranean diet in protecting against several pathologies, especially age-related diseases (cardiovascular diseases, neurodegenerative diseases such as Alzheimer’s disease, eye diseases such as age-related macular degeneration, sarcopenia, and some cancers), via the activities of numerous nutrients with antioxidative and anti-inflammatory activities [29,30,31].

**Figure 2:**
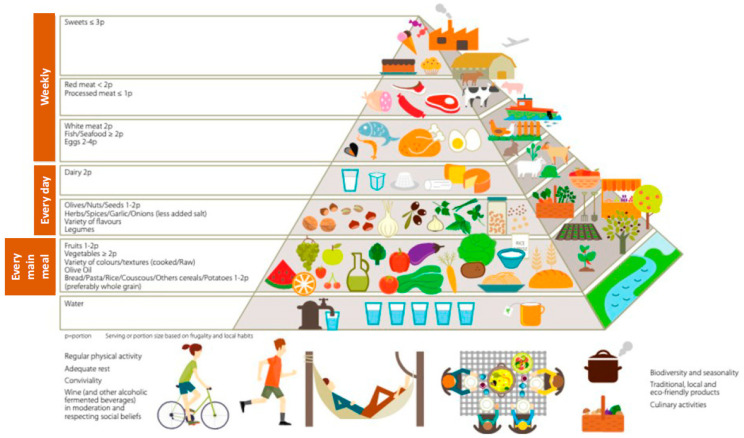
Pyramid of a healthy diet, which characterizes the Mediterranean diet [35].

The Mediterranean diet is based on the famous food pyramid established from the Cretan or Sardinian diet (Figure 2), where the two plant pillars are the olive tree (olive oil) and the vine plants (mainly red wine) [32]. In the cardiovascular field, the Mediterranean diet leads to an 8% reduction in general mortality and a 10% reduction in the risk of developing cardiovascular diseases [33]. Regarding vascular events, the Spanish PREDIMED (Prevention by Mediterranean Diet) study shows that the Mediterranean diet with added olive oil or 30 g/day of nuts reduces the risk of cardiovascular events (heart attack, stroke, and death from cardiovascular diseases) by around 30% [34].

For breast cancer, the adoption of a strict Mediterranean diet is associated with a 30–50% reduction in the incidence of developing breast cancer [36]; for colorectal cancer, the significant association between the strict adoption of a Mediterranean diet and a reduction in the relative risk of developing colorectal cancer is 0.46 [37]. It is estimated that 40% of cancers can be prevented by a healthy diet and a healthy lifestyle (regular walking and no smoking) [38]. When it comes to maintaining brain health, people with good adherence to the Mediterranean diet are 46% less likely to experience a decline in their cognitive abilities, particularly their ability to think [39]. In the longevity register, with a strict Mediterranean diet, leukocytes show the greatest telomere length (*p* = 0.003) [40]. Altogether, these observations support the benefits of the Mediterranean diet and its associated lifestyle with healthy aging [41,42]. 

## 4. Healthy Aging at Mediterranean Latitudes

The impact of diets on healthy aging is crucial. From an economic point of view, healthy aging is a major priority to reduce the cost of aging pathologies, which also have a negative societal impact (https://www.nia.nih.gov/news/social-isolation-loneliness-older-people-pose-health-risks; accessed on 21 January 2025). The urgency to address both population aging and climate change necessitates a rethink and assessment of the impact of climate change on older people [43]. Healthy aging could secondarily favor an increase in longevity. At the moment, in France and Italy, exceptional longevity has been observed for a few people. Examples include Jeanne Calment, aged 122 (France, died 4 August 1997), Robert Marchand, sportsman (cyclist), aged 109 (France, died 20 May 2021), Colette Maze, pianist, aged 108 (France, died November 2023), or the Venice-Padua philosopher Alvise Cornaro (Italy), who advocated a sober lifestyle in the second half of his life and died aged 102 in 1566. All these centenarians followed a low-calorie diet which is also a hallmark of the Mediterranean diet. Elevated percentages of centenarians are also found in Okinawa (Japan), Crete, and Sardinia [34]. In Japan, life expectancy in 2021 was 86 for women and 78 for men. In relation to dietary intake, the longevity of the Japanese population in Okinawa is 10/100,000 inhabitants. Interestingly, this characteristic of longevity and healthy aging does not seem under the control of genetic characteristics but rather of the exposome, including several social and environmental factors impacting physiological processes, which act on aging biology [44]. Thus, among the descendants of these populations who emigrated from Okinawa to Brazil, no more than 2 centenarians/100,000 inhabitants were reported, underlying the importance of environmental factors and diet in aging. In this hybrid Japanese/Brazilian population, energy intake had increased by 30%, which is in line with the Brazilian lifestyle. In the Gers (the south-west region of France), the proportion is 11 centenarians per 100,000, compared with 14 centenarians/per 100,000 in Japan, which has been related to diet habits, especially with the consumption of duck fat reducing the incidence of cardiovascular diseases.

Interestingly, so-called Mediterranean cooking is not restricted to the Mediterranean rim but is also found in other regions of the globe at the same latitude, i.e., between the 39–40th parallels from the northern and southern hemispheres, including California (USA) and certain regions of China, Chile, and South Africa [45].

Interestingly, the Okinawa diet contains similar ingredients to the Mediterranean diet and is known for its major benefits on human health, such as omega-3 fatty acids found in oily fish and edible oils (olive and argan oils, several vitamins and fibers found in fruits and vegetables, as well as several polyphenols with important antioxidant and anti-inflammatory properties found in tea, such as epigallocatechin gallate and resveratrol, which is abundant in red wine) [46]. Consequently, aging and good health do not depend on the population considered but rather on the characteristics of the diet adopted, which strongly impacts aging in good health.

**Figure 3:**
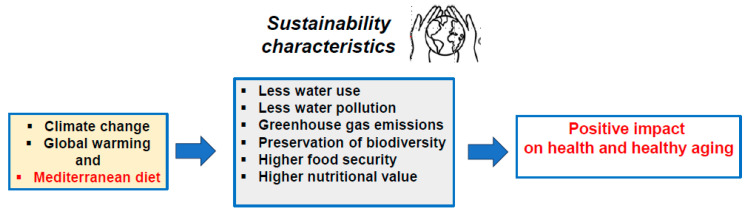
Sustainability features of the Mediterranean diet contributing to its positive ecological footprint.

In terms of nutrition, the alimentation of the future will require several adaptations due to global warming with the simultaneous necessity to reduce carbon footprint; despite these constraints, the food supply of the future will still have to provide enough foods of good nutritional qualities to meet the justified demands of consumers in terms of food safety [47]. The impact of climate change on healthy aging is still not well known. However, the Mediterranean diet is known to have health benefits [48]. Thus, the extension of this diet at higher latitudes is expected to have a positive impact on longevity and good health (Figure 3).

Whether or not environmental conditions will permit the preservation of the benefits of a diet, including some characteristics of the Mediterranean diet, must be considered with caution [11] and will require additional epidemiological studies. While it is crucial to increase adherence to the traditional Mediterranean diet, it is also crucial to reduce the use of pollutants impacting food quality [49]. 

## 5. Climate Change and Modifications of Food Habits

Climate change and media pressure should, in theory, contribute to dietary changes, and these changes should influence a reduction in the global carbon footprint [50]. These changes must be supported by public politics. This applies in particular to meat consumption, which is denounced not only for its environmental impact but also for the often-disastrous living conditions of farm animals. Paradoxically, this does not deter consumers from eating imported food, which often requires extensive irrigation, the financial exploitation of farm workers, and numerous means of transport before arriving on our plates. As a result, virtuous practices are often associated with opposite practices that ultimately fail to have environmental benefits. A more reasoned marketing approach that does not focus solely on financial gain is worth considering.

In the context of climate change, which is being taken into account because of its harmful environmental impact, the notion of consumption summarized by the formula “from fork to farm” is relevant https://www.eea.europa.eu/en/about/contact-us/faqs/how-can-europes-farm-to-fork-strategy-help-achieve-an-environment-friendly-food-system; accessed on 21 January 2025). We need to adapt our diet as much as possible to suit our region and seasonal produce, which does not mean we should stop eating manufactured products. In this context, the use of “Nutriscore” [48], introduced in France, enables consumers to make a choice that takes into account the benefits and harms of the products they choose.

The consequences of climate change for human health are more closely linked to agricultural practices that use pollutant molecules (pesticides, fungicides, fertilizers, antibiotics, hormones, etc.) to optimize plant and animal production in the short and medium term, without taking the long view and the impact this has on human health [51,52]. Since humans are at the end of the food chain, these products end up in our food, with consequences for health at several levels, including fertility, the emergence of new diseases, and an increase in pathologies, neurodegenerative diseases, and cancers.

In the face of climate change, consumer education is a major challenge if we are to respond to the changes in eating habits that are likely to occur. In any case, if changes in eating habits are to occur, they must be made with a view to preserving human health by providing sufficient quality food for all social categories of the population worldwide [53]. The expansion of the Mediterranean climate, with hopefully more Mediterranean foods and ingredients on offer, should benefit the health of the consumers.

Considering the importance of global warming, including heat and hydric stress effects, the preservation of the quality and safety of food, as well as the quantity of food available, will require the development of new technologies to counteract or reduce the impact of climate change [54,55]. 

## 6. Impact on Plant Growth and Characteristics

Throughout the history of the earth, there have been successions of cooling and warming periods associated with major changes in life, including the disappearance, survival, and evolution of species. In the last millennium, in the temperate latitudes of Western Europe, there have been changing cycles of 150–200-year periods in length. The last one started around 100 years ago. For Western Europe, the highest landmarks experiencing changes are the Aletsch glacier in Switzerland and the “Mer de glace” in Chamonix (France) [56]. The area of Bourgogne (Burgundy), France, has also been affected by climate change in the last millennium. Bernard Hudelot, (winemaker and historian from Burgundy, France, 1942–2019) (obituary: https://www.bienpublic.com/edition-cote-de-nuits/2019/08/12/bernard-hudelot-le-pape-des-hautes-cotes-de-nuits, accessed on 22 January 2025) reported in Burgundy alternative cold periods from 1050 to 1200 and from 1200 to 1900 (‘little ice age’).

Thus, it seems that there have always been alternations over several centuries during cooling periods and temperate or heating periods. The present alterations are, however, accentuated by human activities, and to survive, humans have to find rapid solutions. As an example, in terms of culture, the red wine grape varieties in Burgundy are mainly «Pinot noir» in cool periods, and «Gamay» in heat periods and the modification of the cepage does not affect the wine quality.

Michel Magny and Hervé Richard [57] from the area of Bourgogne Franche-Comté (East of France) report the following on global warming: “We realize that things are moving at breakneck speed”. Their book Histoire du climat dans les montagnes du Jura (History of climate in the Jura mountains) is a regional overview of climate issues. It is the result of a compilation of information from 50 French and Swiss contributors. What emerges is the diversity of the types of impact that global warming could have. Michel Magny says the following: “We like the figure that compares the city of Lyon (Middle East of France) to Besançon (North-East of France). In 1980, Besançon caught up with the temperature experienced by Lyon in 1930, only to overtake it today. It’s as if we had traveled 200 km southwards in latitude. Global warming has made us immobile travelers. We’re moving south without moving”.

**Figure 4:**
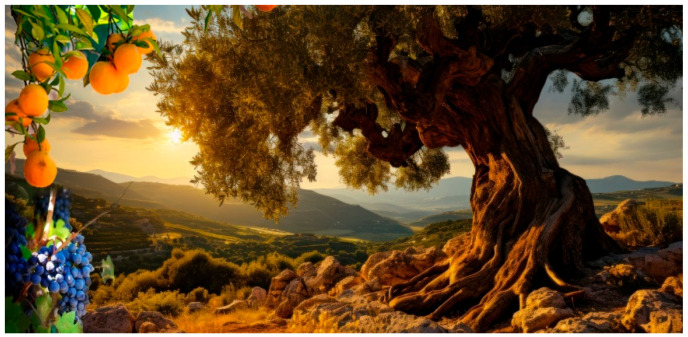
Imaginary landscape of Northern Mediterranean latitudes. With global warming expected in the upcoming years, important changes in Northern Mediterranean latitudes will probably occur and impact the growth of plants and their characteristics (ownership, original picture of the laboratory Bio-PeroxIL, University of Burgundy, Dijon, France).

Consequently, in this current global warming period, we can expect to see a Mediterranean-type climate in the North-East of France, where drought, rainy and windy spells, the disappearance of snow, and heavy frosts will predominate. Besides an actual picture of Burgundy’s landscape with green fields, a wine yard, and trees from temperate regions, we can imagine the appearance of olive trees and agrum trees (Figure 4). The key question will be whether the local products will resist climate change or not. In Burgundy and the Savoy area, grass is already limited to dairy products, which are associated with important industrial activities. The temperature also increases the sugar content in the fruits, including the grapes used to produce wine, which are also associated with important economic activities. Innovative solutions must be found to overcome these problems and have to be considered a challenge.

Thus, in agreement with the observations of Michel Magny and Hervé Richard [57], in 30 years, the Mediterranean climate could move 70 to 100 km north and west in France and soon extend as far as Orléans, which is not far from Paris. We are also observing a gradual shift from four well-marked seasons—spring, summer, autumn, and winter—to two major seasons of unequal length: an increasingly shorter winter that is less cold and less rainy or snowy and a hot summer season with intense rainfalls, thunderstorms, and runoff. The spring and autumn seasons tend to disappear. The climate statistics show the following: rising temperatures: +1.5 °C on average (+0.5 °C on average per decade) and +2.4 °C for May, June, July, and August (+0.8 °C per decade). From May to August, Lyon (Middle East of France) is now hotter than Avignon or Montpellier (South of France) were 30 years ago; an increase in the evaporative capacity of the climate (humidity, air temperature, solar radiation, and wind) of +20 to +25%; a downward trend in rainfall: cumulative rainfall from January to August has fallen by −50 to −60 (−10%); and increasingly frequent and severe droughts (2003, 2005, 2006, 2009, 2011, 2022) that are gradually becoming the norm. The arrival of this Mediterranean climate in our northern regions of France will have a huge impact on both natural vegetation and agriculture, and water resources and plant species’ ability to adapt have not kept pace, hence the development of new varieties that are more resistant to water and heat stress.

Temperature trends are much more marked: temperatures are four times higher in May, June, July, and August than in the winter months, leading to a sharp increase in evapo-sweating, which is a major factor in agricultural production. As a result, aridity is becoming increasingly pronounced, leading to droughts that jeopardize harvests (cereals, forage) and cause fires that modify the landscape [58]. We are at the very beginning of the climate change caused by our massive greenhouse gas emissions (see the report by the IPCC (Inter-governmental Panel on Climate Change) (https://www.llnl.gov/article/47871/ipcc-reports-climate-change-widespread-rapid-intensifying; accessed on 22 January 2025) and the repercussions are not only global but also local. Integrated solutions for global and local climate change are rapidly required and must be adapted to the dynamic climate reality [59]. 

## 7. Perspectives Given by Experts

**Figure 5:**
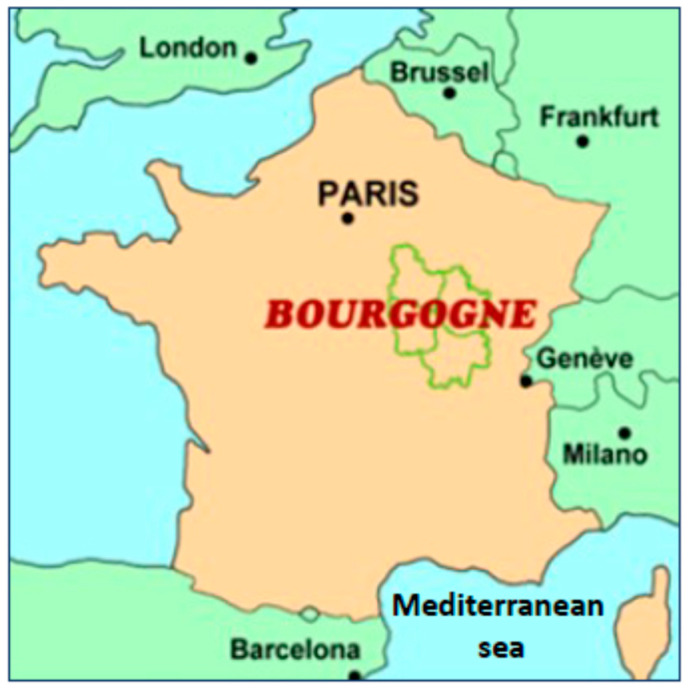
Map of Burgundy (Bourgogne), a region in France located south at 500 km of the Mediterranean Sea (free access illustration; Google; modified by G. Lizard).

The climate projection for Burgundy (Bourgogne) in the next 50 years and its consequences for vines and food, with the role of climate disruption and the potential development of a Mediterranean diet, represents an important field of study at the University of Burgundy (Bourgogne) in Dijon, France) (Figure 5).

The climatologists from Dijon (Burgundy) suggest the existence of a safe limit below 2 °C of global warming for the European winemaking sector (including Burgundy), while adaptation might become far more challenging beyond this threshold [60]. There are several arguments supporting the fact that climate change will not dramatically decrease viticultural suitability in the main wine-producing areas in France by 2050 [61]. The cultivation of 400 varieties of tomatoes using the methods of past gardeners is one aspect of ongoing experimentation in the garden in the face of climate change. Ongoing climate change (rising temperatures, winds, and irregular and abundant rainfall) is having a profound impact on plant growth in the vegetable garden. The concept of climate change is very anxiety-provoking, but when it comes to gardening, the rapid changes underway should lead us to operate differently and in a positive way [62,63]. Indeed, while climate change disrupts certain crops, it also allows others to flourish. Summer is becoming a very difficult season for many vegetables, but the three other seasons are opening up new possibilities that have been unknown until now. It is now easier than ever to have fresh vegetables all year. In agreement with these observations in Burgundy and the East of France, several works report that gardening can provide solutions to face climate change in different world regions [64]. Efforts will result in the introduction of new crops, new gardening practices, and new ways of eating and cooking, leading to the consumption of seasonal fruits and vegetables and the use of short distribution channels using local production for a reduction in carbon footprint (circular economy) [65,66]. In gardening and agriculture, it is, however, known whether the soil microbiome governs the biogeochemical cycling of macronutrients, micronutrients, and other elements vital for the growth of plants and animal life. A better understanding and better control of soil microorganisms could help mitigate the negative consequences of climate change [67].

The rise in sustainable food and its media coverage in recent years has led to a paradigmatic shift from eating to eating well, resulting in changes in public policies and social representations. This research is being carried out as part of the implementation of a territorial policy for Dijon (France), a medium-sized French metropolis, to highlight the political and communicational dimensions of sustainable food in response to contemporary climate issues. The leitmotiv is “Less meat, more proteinaceous: political, climatic and communicational issues” [68]. This assumption is not shared by those who consider it unreasonable to dissuade consumers from eating meat to decrease their carbon footprint and stop intensive farming in favor of extensive farming. An evolution towards a Mediterranean-style diet and its beneficial effects on carbon footprint and health have been reported in several studies. The development of the Mediterranean diet will favor a decrease in carbon footprint [69], whereas a great diversity will probably persist among the countries concerned. The most important carbon footprint is reported for cereals and bovine meat [70]. Acting at this level, with international and local actions, therefore, seems essential. Reduced consumption of red meat and ultra-processed foods realized with red meat will not only decrease carbon footprint but also the price of meals with a beneficial economic impact on the consumer [71,72,73,74]. To obtain protein-rich proteinaceous foods, conventional cultivation techniques, with a low environmental impact and supporting local agriculture will be preferred to industrial agriculture, with a high carbon impact and sometimes questionable nutritional qualities. In those conditions, if lower prices are associated with food quality and lower carbon footprint, the consumer will easily move towards the Mediterranean diet and progressively change their diet habits. Understanding individual food choices and the integration of climate change is required to transform the current food system to ensure the health of people and the sustainability of the planet [75]. 

## 8. The Adaptation of Populations to Agriculture and Food and Cooking Changes in the Mediterranean Diet 

Climate change will undoubtedly lead to changes in crop production across the globe, with periods of rainfall and drought being severely disrupted [73]. In the short term, this may result in a reduction in the production of certain basic products that are essential for feeding people and the animals they consume, leading to an increase in the cost of basic products and, more or less serious, socio-economic problems. To remedy this situation, we need to develop innovative crop and livestock farming techniques. Limiting the impact of climate change on agricultural production is vital for the future of humanity [74,76].

Another aspect is the energy required to produce food raw materials and the processed products derived from them [77]. It is highly recommended to limit transport, produce locally (to reduce food packaging), and decrease fossil fuel consumption [78]. This will also contribute to reducing waste, especially that resulting from the packaging [79]. In the agri-food industry, the energy required for production lines will have to be reduced, and packaging optimized to adapt consumption to individual needs without encouraging over-consumption, which is a cause of many illnesses, particularly cardiovascular diseases, diabetes, and metabolic syndrome [80].

Finally, at the consumer level, the latter are looking for food that is quick, not expensive, and easy to eat daily (such as street food) and that consumes little energy. A shift in culinary practices is taking place [81], supported by major industrial groups in the food industry, which propose cooking appliances that can be used to produce classic, high-quality recipes with reduced energy consumption. The expansion of the Mediterranean climate, with consequently more Mediterranean foods and ingredients, should be beneficial for the health of consumers.

It is noteworthy that faced with climate change, the concept of planetary nutrition or the so-called “Planeterranean diet” has emerged and plays an important part in the Mediterranean diet [82], characterized by food diversity and the high content of antioxidants [83,84]. This new concept can be considered to have continued progression in some parts of the world, including Northern Mediterranean latitudes, except in particular places (deserts, arid regions, and high mountains). It is, however, important to underline the fact that the Mediterranean diet is not just a pattern of food to be consumed; it must also be contextualized in the social background of the Mediterranean culture. While the methodologies to study the Mediterranean diet have been demonstrated to be useful until now, a more holistic approach should be considered in future studies to be applied globally through the concept of a “Planeterranean diet” [82].

## 9. Limitations and Future Directions

In the near future, besides economic interests, climate change will favor the consumption of several products associated with the Mediterranean diet in Northern European latitudes. In this context, the roles of producers and consumers are major in finding the best agreement. 

Future investigations will have to determine the impact of the progression of the Mediterranean climate in the highest latitudes of Europe on the incidence of (i) health, plant growth, farming, and food habits; (ii) the development of new farming practices with less environmental impacts, and (iii) societal adaptations of populations towards agriculture, food, and cooking changes.

## 10. Conclusions

Climate change, which favors the progression of the Mediterranean climate in the highest latitudes of Europe, has a progressively important impact on agricultural practices and eating behaviors. This narrative review shows that several thoughts and actions need to be realized to minimize the social and economic impact of global warming, which will also lead to several innovations in many domains: health, farming, and eating habits. Whereas climate change affects all areas of the world, including the Burgundy (Bourgogne) area in France, which has a lot of traditions in nutrition and cooking habits, the challenge of this French area shall be to continue to provide high-quality food products, such as cereal, meat, cheese, and wine, which is also a part of Mediterranean-diet. 

As eating behaviors differ between regions within the same country, and even more from one country to another, the measures implemented to guarantee food security will also have to take dietary diversity into account. There is, therefore, a considerable challenge for the agriculture and food industry to deal with climate change in the agri-food sector, taking into account nutritional and cultural aspects, and effort is also required from the consumer to adapt their food habits. 

## References

[1] McElroy M.B. (1994). Climate of the earth: An overview. Environ. Pollut..

[2] Rousseau D.-D., Bagniewski W., Lucarini V. (2023). A punctuated equilibrium analysis of the climate evolution of cenozoic exhibits a hierarchy of abrupt transitions. Sci. Rep..

[3] Nikendei C., Bugaj T.J., Nikendei F., Kühl S.J., Kühl M. (2020). Klimawandel: Ursachen, Folgen, Lösungsansätze und Implikationen für das Gesundheitswesen [Climate change: Causes, consequences, solutions and public health care implications]. Z. Evidenz Fortbild. Qual. Gesundheitswesen.

[4] Morales-Castilla I., de Cortázar-Atauri I.G., Cook B.I., Lacombe T., Parker A., van Leeuwen C., Nicholas K.A., Wolkovich E.M. (2020). Diversity buffers winegrowing regions from climate change losses. Proc. Natl. Acad. Sci. USA.

[5] Tierney J.E., Poulsen C.J., Montañez I.P., Bhattacharya T., Feng R., Ford H.L., Hönisch B., Inglis G.N., Petersen S.V., Sagoo N. (2020). Past climates inform our future. Science.

[6] Fordham D.A., Jackson S.T., Brown S.C., Huntley B., Brook B.W., Dahl-Jensen D., Gilbert M.T.P., Otto-Bliesner B.L., Svensson A., Theodoridis S. (2020). Using paleo-archives to safeguard biodiversity under climate change. Science.

[7] Aboul-Enein B.H., Puddy W.C., Bernstein J. (2020). Ancel Benjamin Keys (1904–2004): His early works and the legacy of the modern Mediterranean diet. J. Med. Biogr..

[8] Renaud S., de Lorgeril M. (1992). Wine, alcohol, platelets, and the French paradox for coronary heart disease. Lancet.

[9] Martini D. (2019). Health Benefits of Mediterranean Diet. Nutrients.

[10] Romanello M., Walawender M., Hsu S.-C., Moskeland A., Palmeiro-Silva Y., Scamman D., Ali Z., Ameli N., Angelova D., Ayeb-Karlsson S. (2024). The 2024 report of the Lancet Countdown on health and climate change: Facing record-breaking threats from delayed action. Lancet.

[11] Dixon K.A., Michelsen M.K., Carpenter C.L. (2023). Modern Diets and the Health of Our Planet: An Investigation into the Environmental Impacts of Food Choices. Nutrients.

[12] Radd-Vagenas S., Kouris-Blazos A., Singh M.F., Flood V.M. (2017). Evolution of Mediterranean diets and cuisine: Concepts and definitions. Asia Pac. J. Clin. Nutr..

[13] Rishor-Olney C.R., Hinson M.R. (2023). Mediterranean Diet.

[14] Trichopoulou A., Martínez-González M.A., Tong T.Y., Forouhi N.G., Khandelwal S., Prabhakaran D., Mozaffarian D., de Lorgeril M. (2014). Definitions and potential health benefits of the Mediterranean diet: Views from experts around the world. BMC Med..

[15] Damigou E., Faka A., Kouvari M., Anastasiou C., Kosti R.I., Chalkias C., Panagiotakos D. (2023). Adherence to a Mediterranean type of diet in the world: A geographical analysis based on a systematic review of 57 studies with 1,125,560 participants. Int. J. Food Sci. Nutr..

[16] Xing Y., Wang X. (2024). Precision Agriculture and Water Conservation Strategies for Sustainable Crop Production in Arid Regions. Plants.

[17] Martins F.P., Paschoalotto M.A.C., Closs J., Bukowski M., Veras M.M. (2024). The Double Burden: Climate Change Challenges for Health Systems. Environ. Health Insights.

[18] Habib-Ur-Rahman M., Ahmad A., Raza A., Hasnain M.U., Alharby H.F., Alzahrani Y.M., Bamagoos A.A., Hakeem K.R., Ahmad S., Nasim W. (2022). Impact of climate change on agricultural production; Issues, challenges, and opportunities in Asia. Front. Plant Sci..

[19] Mukherjee P.K., Das B., Bhardwaj P.K., Tampha S., Singh H.K., Chanu L.D., Sharma N., Devi S.I. (2023). Socio-economic sustainability with circular economy—An alternative approach. Sci. Total Environ..

[20] NMS Association Nutrition Méditerranéenne et Santé #W832013271. https://anms.e-monsite.com/pages/les-actions.html.

[21] Lins M., Puppin Zandonadi R., Raposo A., Ginani V. (2021). Food Waste on Foodservice: An Overview through the Perspective of Sustainable Dimensions. Foods.

[22] Guasch-Ferré M., Willett W.C. (2021). The Mediterranean diet and health: A comprehensive overview. J. Intern. Med..

[23] Sikalidis A.K., Kelleher A.H., Kristo A.S. (2021). Mediterranean Diet. Encyclopedia.

[24] Shannon O.M., Ashor A.W., Scialo F., Saretzki G., Martin-Ruiz C., Lara J., Matu J., Griffiths A., Robinson N., Lillà L. (2021). Mediterranean diet and the hallmarks of ageing. Eur. J. Clin. Nutr..

[25] Laster J., Frame L.A. (2019). Beyond the calories-is the problem in the processing?. Curr. Treat Options Gastroenterol..

[26] Salehi B., Quispe C., Sharifi-Rad J., Cruz-Martins N., Nigam M., Mishra A.P., Konovalov D.A., Orobinskaya V., Abu-Reidah I.M., Zam W. (2021). Phytosterols: From Preclinical Evidence to Potential Clinical Applications. Front. Pharmacol..

[27] Furman D., Campisi J., Verdin E., Carrera-Bastos P., Targ S., Franceschi C., Ferrucci L., Gilroy D.W., Fasano A., Miller G.W. (2019). Chronic inflammation in the etiology of disease across the life span. Nat. Med..

[28] Trichopoulou A. (2021). Mediterranean diet as intangible heritage of humanity: 10 years on. Nutr. Metab. Cardiovasc. Dis..

[29] Kessas K., Chouari Z., Ghzaiel I., Zarrouk A., Ksila M., Ghrairi T., El Midaoui A., Lizard G., Kharoubi O. (2022). Role of Bioactive Compounds in the Regulation of Mitochondrial Dysfunctions in Brain and Age-Related Neurodegenerative Diseases. Cells.

[30] Rezig L., Ghzaiel I., Ksila M., Yammine A., Nury T., Zarrouk A., Samadi M., Chouaibi M., Vejux A., Lizard G. (2022). Cytoprotective activities of representative nutrients from the Mediterranean diet and of Mediterranean oils against 7-ketocholesterol- and 7β-hydroxycholesterol-induced cytotoxicity: Application to age-related diseases and civilization diseases. Steroids.

[31] Zhor C., Wafaa L., Ghzaiel I., Kessas K., Zarrouk A., Ksila M., Ghrairi T., Latruffe N., Masmoudi-Kouki O., El Midaoui A. (2023). Effects of polyphenols and their metabolites on age-related diseases. Biochem. Pharmacol..

[32] Carluccio M.A., Siculella L., Ancora M.A., Massaro M., Scoditti E., Storelli C., Visioli F., Distante A., De Caterina R. (2003). Olive oil and red wine antioxidant polyphenols inhibit endothelial activation: Antiatherogenic properties of Mediterranean diet phytochemicals. Arterioscler. Thromb. Vasc. Biol..

[33] Mora C., McKenzie T., Gaw I.M., Dean J.M., von Hammerstein H., Knudson T.A., Setter R.O., Smith C.Z., Webster K.M., Patz J.A. (2022). Over half of known human pathogenic diseases can be aggravated by climate change. Nat. Clim. Change.

[34] Laffond A., Rivera-Picón C., Rodríguez-Muñoz P.M., Juárez-Vela R., Ruiz de Viñaspre-Hernández R., Navas-Echazarreta N., Sánchez-González J.L. (2023). Mediterranean Diet for Primary and Secondary Prevention of Cardiovascular Disease and Mortality: An Updated Systematic Review. Nutrients.

[35] Serra-Majem L., Tomaino L., Dernini S., Berry E.M., Lairon D., Ngo de la Cruz J., Bach-Faig A., Donini L.M., Medina F.-X., Belahsen R. (2020). Updating the Mediterranean Diet Pyramid towards Sustainability: Focus on Environmental Concerns. Int. J. Environ. Res. Public Health.

[36] Villarini M., Lanari C., Nucci D., Gianfredi V., Marzulli T., Berrino F., Borgo A., Bruno E., Gargano G., Moretti M. (2016). Community-based participatory research to improve life quality and clinical outcomes of patients with breast cancer (DianaWeb in Umbria pilot study). BMJ Open.

[37] Grosso G., Biondi A., Galvano F., Mistretta A., Marventano S., Buscemi S., Drago F., Basile F. (2014). Factors associated with colorectal cancer in the context of the Mediterranean diet: A case-control study. Nutr. Cancer.

[38] Yammine A., Namsi A., Vervandier-Fasseur D., Mackrill J.J., Lizard G., Latruffe N. (2021). Polyphenols of the Mediterranean Diet and Their Metabolites in the Prevention of Colorectal Cancer. Molecules.

[39] McEvoy C.T., Hoang T., Sidney S., Steffen L.M., Jacobs D.R., Shikany J.M., Wilkins J.T., Yaffe K. (2019). Dietary patterns during adulthood and cognitive performance in midlife: The CARDIA study. Neurology.

[40] Boccardi V., Esposito A., Rizzo M.R., Marfella R., Barbieri M., Paolisso G. (2013). Mediterranean diet, telomere maintenance and health status among elderly. PLoS ONE.

[41] Clegg M.E., Williams E.A. (2018). Optimizing nutrition in older people. Maturitas.

[42] Dobroslavska P., Silva M.L., Vicente. F., Pereira P. (2024). Mediterranean Dietary Pattern for Healthy and Active Aging: A Narrative Review of an Integrative and Sustainable Approach. Nutrients.

[43] Prina M., Khan N., Khan S.A., Caicedo J.C., Peycheva A., Seo V., Xue S., Sadana R. (2024). Climate change and healthy ageing: An assessment of the impact of climate hazards on older people. J. Glob. Health.

[44] Nielsen L., Marsland A.L., Hamlat E.J., Epel E.S. (2024). New Directions in Geroscience: Integrating Social and Behavioral Drivers of Biological Aging. Psychosom. Med..

[45] Martínez-González M.Á., Hershey M.S., Zazpe I., Trichopoulou A. (2017). Transferability of the Mediterranean Diet to Non-Mediterranean Countries. What Is and What Is Not the Mediterranean Diet. Nutrients.

[46] Pes G.M., Dore M.P., Tsofliou F., Poulain M. (2022). Diet and longevity in the Blue Zones: A set-and-forget issue?. Maturitas.

[47] Duchenne-Moutien R.A., Neetoo H. (2021). Climate Change and Emerging Food Safety Issues: A Review. J. Food Prot..

[48] Hercberg S., Touvier M., Salas-Salvado J., Group of European scientists supporting the implementation of Nutri-Score in Europe (2022). The Nutri-Score nutrition label. Int. J. Vitam. Nutr. Res..

[49] Lacirignola C., Capone R., Debs P., El Bilali H., Bottalico F. (2014). Natural resources–Food nexus: Food-related environmental footprints in the mediterranean countries. Front. Nutr..

[50] Li Y., He P., Shan Y., Li Y., Hang Y., Shao S., Ruzzenenti F., Hubacek K. (2024). Reducing climate change impacts from the global food system through diet shifts. Nat. Clim. Chang..

[51] Boxall A.B., Hardy A., Beulke S., Boucard T., Burgin L., Falloon P.D., Haygarth P.M., Hutchinson T., Kovats R.S., Leonardi G. (2009). Impacts of climate change on indirect human exposure to pathogens and chemicals from agriculture. Environ. Health Perspect..

[52] Hassaan M.A., El Nemr A. (2020). Pesticides pollution: Classifications, human health impact, extraction and treatment techniques. Egypt. J. Aquat. Res..

[53] Koliaki C.C., Katsilambros N.L., Dimosthenopoulos C. (2024). The Mediterranean Diet in the Era of Climate Change: A Reference Diet for Human and Planetary Health. Climate.

[54] Alam A.N., Lee E.Y., Hossain M.J., Samad A., Kim S.H., Hwang Y.H., Joo S.T. (2024). Meat quality and safety issues during high temperatures and cutting-edge technologies to mitigate the scenario. J. Anim. Sci. Technol..

[55] Hoffman L.C., Schreuder J., Cozzolino D. (2024). Food authenticity and the interactions with human health and climate change. Crit. Rev. Food Sci. Nutr..

[56] Holtzhauser H. (1984). Zur Geschichte der Aletschgletscher und des Fieschergletschers.

[57] Magny M., Richard H. (2023). Climate History in Jura Mountains.

[58] Miralles D.G., Gentine P., Seneviratne S.I., Teuling A.J. (2019). Land-atmospheric feedbacks during droughts and heatwaves: State of the science and current challenges. Ann. N. Y. Acad. Sci..

[59] Lin B.B., Ossola A., Alberti M., Andersson E., Bai X., Dobbs C., Elmqvist T., Evans K.L., Frantzeskaki N., Fuller R.A. (2021). Integrating solutions to adapt cities for climate change. Lancet Planet. Health.

[60] Sgubin G., Swingedouw D., Mignot J., Gambetta G.A., Bois B., Loukos H., Noël T., Pieri P., de Cortázar-Atauri I.G., Ollat N. (2023). Non-linear loss of suitable wine regions over Europe in response to increasing global warming. Glob. Change Biol..

[61] van Leeuwen C., Schultz H.R., de Cortazar-Atauri I.G., Duchêne E., Ollat N., Pieri P., Bois B., Goutouly J.-P., Quénol H., Touzard J.-M. (2013). Why climate change will not dramatically decrease viticultural suitability in main wine-producing areas by 2050. Proc. Natl. Acad. Sci. USA.

[62] Matías J., Rodríguez M.J., Carrillo-Vico A., Casals J., Fondevilla S., Haros C.M., Pedroche J., Aparicio N., Fernández-García N., Aguiló-Aguayo I. (2024). From ‘Farm to Fork’: Exploring the Potential of Nutrient-Rich and Stress-Resilient Emergent Crops for Sustainable and Healthy Food in the Mediterranean Region in the Face of Climate Change Challenges. Plants.

[63] Balestrazzi A., Calvio C., Macovei A., Pagano A., Laux P., Moutahir H., Rajjou L., Tani E., Chachalis D., Katsis C. (2024). Seed quality as a proxy of climate-ready orphan legumes: The need for a multidisciplinary and multi-actor vision. Front. Plant Sci..

[64] Larran A.S., Pajoro A., Qüesta J.I. (2023). Is winter coming? Impact of the changing climate on plant responses to cold temperature. Plant Cell Environ..

[65] Bajaj K., Mehrabi Z., Kastner T., Jägermeyr J., Müller C., Schwarzmüller F., Hertel T.W., Ramankutty N. (2025). Current food trade helps mitigate future climate change impacts in lower-income nations. PLoS ONE.

[66] Cai C., Lv L., Wei S., Zhang L., Cao W. (2024). How does climate change affect potential yields of four staple grain crops worldwide by 2030?. PLoS ONE.

[67] Jansson J.K., Hofmockel K.S. (2020). Soil microbiomes and climate change. Nat. Rev. Microbiol..

[68] Castaldi S., Dembska K., Antonelli M., Petersson T., Piccolo M.G., Valentini R. (2022). The positive climate impact of the Mediterranean diet and current divergence of Mediterranean countries towards less climate sustainable food consumption patterns. Sci. Rep..

[69] Unar-Munguía M., Cervantes-Armenta M.A., Rodríguez-Ramírez S., Arenas A.B., Gaxiola A.C.F., Rivera J.A. (2024). Mexican national dietary guidelines promote less costly and environmentally sustainable diets. Nat. Food.

[70] Stylianou K.S., Fulgoni V.L., Jolliet O. (2021). Small targeted dietary changes can yield substantial gains for human health and the environment. Nat. Food.

[71] Khazaei H., Subedi M., Nickerson M., Martínez-Villaluenga C., Frias J., Vandenberg A. (2019). Seed Protein of Lentils: Current Status, Progress, and Food Applications. Foods.

[72] Begum N., Khan Q.U., Liu L.G., Li W., Liu D., Haq I.U. (2023). Nutritional composition, health benefits and bio-active compounds of chickpea (*Cicer arietinum* L.). Front. Nutr..

[73] Verma K.K., Song X., Kumari A., Jagadesh M., Singh S.K., Bhatt R., Singh M., Seth C.S., Li Y. (2024). Climate change adaptation: Challenges for agricultural sustainability. Plant Cell Environ..

[74] Campa M., Miranda S., Licciardello C., Lashbrooke J.G., Costa L.D., Guan Q., Spök A., Malnoy M. (2023). Application of new breeding techniques in fruit trees. Plant Physiol..

[75] Chen P.J., Antonelli M. (2020). Conceptual Models of Food Choice: Influential Factors Related to Foods, Individual Differences, and Society. Foods.

[76] Pixley K.V., Cairns J.E., Lopez-Ridaura S., Ojiewo C.O., Dawud M.A., Drabo I., Mindaye T., Nebie B., Asea G., Das B. (2023). Redesigning crop varieties to win the race between climate change and food security. Mol. Plant.

[77] Ready E., Ross C.T., Beheim B., Parrott J. (2024). Indigenous food production in a carbon economy. Proc. Natl. Acad. Sci. USA.

[78] Cruz R.M.S., Albertos I., Romero J., Agriopoulou S., Varzakas T. (2024). Innovations in Food Packaging for a Sustainable and Circular Economy. Adv. Food Nutr. Res..

[79] Anuardo R.G., Espuny M., Costa A.C.F., Oliveira O.J. (2022). Toward a cleaner and more sustainable world: A framework to develop and improve waste management through organizations, governments and academia. Heliyon.

[80] Ramkumar D., Marty A., Ramkumar J., Rosencranz H., Vedantham R., Goldman M., Meyer E., Steinmetz J., Weckle A., Bloedorn K. (2024). Food for thought: Making the case for food produced via regenerative agriculture in the battle against non-communicable chronic diseases (NCDs). One Health.

[81] Habib R., White K., Hardisty D.J., Zhao J. (2021). Shifting consumer behavior to address climate change. Curr. Opin. Psychol..

[82] Godos J., Scazzina F., Castello C.P., Giampieri F., Quiles J.L., Urbano M.B., Battino M., Galvano F., Iacoviello L., de Gaetano G. (2024). Underrated aspects of a true Mediterranean diet: Understanding traditional features for worldwide application of a “Planeterranean” diet. J. Transl. Med..

[83] Singh R.B., Fedacko J., Fatima G., Magomedova A., Watanabe S., Elkilany G. (2022). Why and How the Indo-Mediterranean Diet May Be Superior to Other Diets: The Role of Antioxidants in the Diet. Nutrients.

[84] Deleu S., Becherucci G., Godny L., Mentella M.C., Petito V., Scaldaferri F. (2024). The Key Nutrients in the Mediterranean Diet and Their Effects in Inflammatory Bowel Disease: A Narrative Review. Nutrients.

